# Vigilance Task-Related Change in Brain Functional Connectivity as Revealed by Wavelet Phase Coherence Analysis of Near-Infrared Spectroscopy Signals

**DOI:** 10.3389/fnhum.2016.00400

**Published:** 2016-08-05

**Authors:** Wei Wang, Bitian Wang, Lingguo Bu, Liwei Xu, Zengyong Li, Yubo Fan

**Affiliations:** ^1^Key Laboratory of High Efficiency and Clean Mechanical Manufacture, School of Mechanical Engineering, Shandong UniversityJinan, China; ^2^National Research Center for Rehabilitation Technical AidsBeijing, China

**Keywords:** near-infrared spectroscopy, attention, functional connectivity, global connectivity, wavelet phase coherence

## Abstract

This study aims to assess the vigilance task-related change in connectivity in healthy adults using wavelet phase coherence (WPCO) analysis of near-infrared spectroscopy signals (NIRS). NIRS is a non-invasive neuroimaging technique for assessing brain activity. Continuous recordings of the NIRS signals were obtained from the prefrontal cortex (PFC) and sensorimotor cortical areas of 20 young healthy adults (24.9 ± 3.3 years) during a 10-min resting state and a 20-min vigilance task state. The vigilance task was used to simulate driving mental load by judging three random numbers (i.e., whether odd numbers). The task was divided into two sessions: the first 10 min (Task t1) and the second 10 min (Task t2). The WPCO of six channel pairs were calculated in five frequency intervals: 0.6–2 Hz (I), 0.145–0.6 Hz (II), 0.052–0.145 Hz (III), 0.021–0.052 Hz (IV), and 0.0095–0.021 Hz (V). The significant WPCO formed global connectivity (GC) maps in intervals I and II and functional connectivity (FC) maps in intervals III to V. Results show that the GC levels in interval I and FC levels in interval III were significantly lower in the Task t2 than in the resting state (*p* < 0.05), particularly between the left PFC and bilateral sensorimotor regions. Also, the reaction time (RT) shows an increase in Task t2 compared with that in Task t1. However, no significant difference in WPCO was found between Task t1 and resting state. The results showed that the change in FC at the range of 0.6–2 Hz was not attributed to the vigilance task *per se*, but the interaction effect of vigilance task and time factors. The findings suggest that the decreased attention level might be partly attributed to the reduced GC levels between the left prefrontal region and sensorimotor area. The present results provide a new insight into the vigilance task-related brain activity.

## Introduction

A high-level attention cannot constantly be maintained during a cognitively demanding task, such as driving; the level of attention progressively diminishes as time elapses, thereby affecting task performance negatively (Derosière et al., 2014). Attention decrements may lead to work-related injuries (Grandjean, 1979; Czeisler et al., 2005) and traffic accidents (Lal and Craig, 2001; Beede and Kass, 2006). Driving a vehicle requires the ability to make decisions, planning, and visual attention. Driver attention level decreases after a long time of driving. Understanding the cognitive state of a driver in real-time can assist in the design of in-vehicle interfaces. Therefore, clarifying the relationship between driver attention state and brain activity, including recognition and judgment, is necessary.

Resting state functional connectivity (RSFC) is characterized by a temporal correlation between two time series in low frequency (0–0.1 Hz; Biswal et al., 1995). RSFC has been extensively studied within the related brain regions, including the sensory, motor, sensory association, attention, and task-negative regions, using functional magnetic resonance imaging (fMRI; Fox and Raichle, 2007), near-infrared spectroscopy (NIRS; Li et al., 2010; Lu et al., 2010; Sasai et al., 2011), electroencephalography (EEG) or magnetoencephalography (MEG; Aihara et al., 2012; Scholkmann et al., 2014). The RSFC maps reflect the intrinsic functional architecture of the human brain (Lu et al., 2010). The correlation of brain activity in the functional connectivity (FC) maps can provide insights into the intrinsic functional architecture of the human brain networks (Biswal et al., 1995; Lu et al., 2010).

NIRS is an optical neuroimaging method that has been extensively adopted to detect real-time changes of brain activity. NIRS is a non-invasive method that continuously monitors brain activity by measuring the absorption of near-infrared light through the intact skull (Villringer and Dirnagl, 1994). NIRS has unique features compared with other non-invasive brain imaging techniques (i.e., fMRI), such as data quality (i.e., temporal and spatial resolutions) and logistics (i.e., cost, equipment portability, and patient compatibility; Lu et al., 2010; Tan et al., 2015).

Studies using NIRS has successfully observed FC in adult and infant participants (Taga et al., 2000; Sasai et al., 2011; Ferrari and Quaresima, 2012). The high FC over an extensive range (0.009–0.1 Hz) has been determined between the homologous cortical regions of the contralateral hemisphere (i.e., homologous connectivity) based on the oxy-Hb signals (Sasai et al., 2011). Also, NIRS has been used to detect changes in the FC response to cognitive load task and task-free state and these results demonstrate that NIRS is sensitive to both cognitive load and state (Derosière et al., 2014; Fishburn et al., 2014).

The NIRS signals obtained from the cortical regions during the resting state mainly reflect regional hemodynamic fluctuations that originated from spontaneous cortical activity (Ferrari and Quaresima, 2012). Sasai et al. (2011) used multichannel NIRS to investigate frequency dependency of FC between different regions by decomposing fluctuations of oxy-Hb and deoxy-Hb signals into various frequency bands. The NIRS signals comprise different features in the time and frequency domains (Bajaj et al., 2014). The quantification of the NIRS information can be estimated using the Hilbert transform, Fourier transform, or wavelet transform, which are mathematically equivalent when applied in spectral analyses (Bruns, 2004). However, the Fourier transform fails in providing good time and frequency resolutions in low or high frequencies. In contrast, wavelet transform using the Morlet wavelet provides an adjustable window, thereby offering an intuitive visualization of the time–frequency domain and high resolution in both high and low frequency components (Stefanovska et al., 1999; Han et al., 2014b). The approach used for NIRS signals based on wavelet has been used to reveal frequency-specific FC in elderly subjects with cerebral infarction (CI; Tan et al., 2015). Characteristic frequencies of cerebral oxy-Hb signals corresponding to specific origins have been identified using wavelet analysis (Li et al., 2010; Tan et al., 2015, 2016).

Wavelet phase coherence (WPCO) can reveal a possible relationship by evaluating the match between the instantaneous phases of two signals (Bandrivskyy et al., 2004; Bernjak et al., 2012). WPCO is particularly valuable for low frequency components, thereby significantly contributing to the cardiovascular system (Bernjak et al., 2012). Wavelet-based analysis has been performed to detect relationships among skin blood flows, temperature, cerebral oxygenation and blood pressure within certain frequency ranges (Bandrivskyy et al., 2004; Bernjak et al., 2012; Gao et al., 2015; Tan et al., 2015).

The prefrontal cortex (PFC) plays an important role in cognitive control (Miller and Cohen, 2001; Derosière et al., 2014). Sensorimotor cortex located anterior and posterior of the brain central sulcus, and is crucial in sensation and motor control (Franceschini et al., 2003). Attention lapses were demonstrated to happen in the different cortical regions (Weissman et al., 2006). However, little information is known about the changes in the vigilance task-related FC maps in characteristic frequencies ranges. In this study, we hypothesized that the vigilance task would induce significant change in the connectivity maps in characteristic frequency bands in the PFC and sensorimotor areas. Therefore, the objective of this study was to evaluate the change in vigilance task-related connectivity in the prefrontal–sensorimotor regions using the WPCO methods. The present results would provide a new insight into the driving ability and facilitating the prevention of vigilance task-related accidents.

## Materials and Methods

### Subjects

A total of 20 young healthy subjects (aged: 24.9 ± 3.3 years) including 8 males and 12 females were recruited from the university to participate in this study. The participant should have a valid driving license and enough sleep time (no less than 7 h). The participants were not under psychotropic medication (e.g., stimulants, anti-depressants, and anxiolytics) and had no history of neurological injury or disease, seizure disorder, or psychiatric diagnosis. All the participants were right-handed and had normal or corrected vision. The subjects reported their physical information and amount of sleep on the night before the experiment was conducted. Basic information of the participants, including their age, weight, height, and blood pressure, was recorded before the experiment. The subjects were prohibited from having sensitive drinks (i.e., alcohol or caffeine) 12 h prior to the experimental testing. All the participants provided written informed consent before participating in the study. The subjects were instructed to be familiar with the protocol prior to the experiment. The experimental procedure was approved by the Human Ethics Committee of Shandong University and was in accordance with the ethical standards specified by the Helsinki Declaration of 1975 (revised in 1983).

### Task Procedure

**Figure 1:**
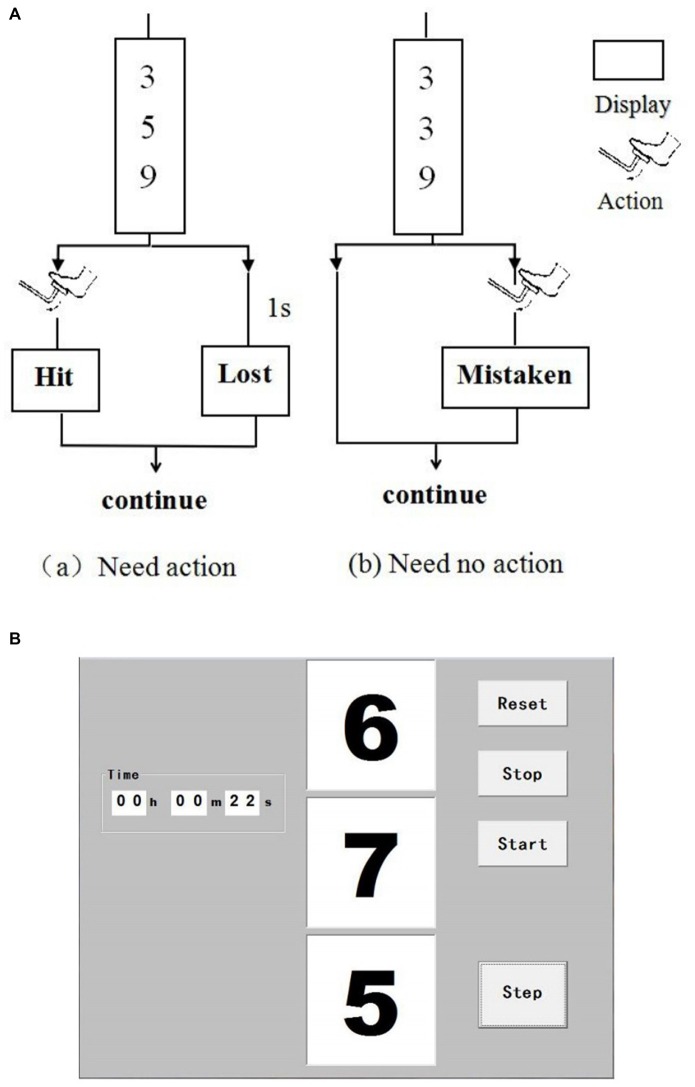
**Interface of the vigilance task.** Three numbers change randomly per second on the screen **(A)**. Subjects need to step on the brake pedal (i.e., click the “Step” button on the screen within 1 s) when the three numbers are different odd ones **(a)**. Otherwise, the subjects do not need to react **(b)**. **(B)** Shows the interface of the software when operating.

Driving is in high-vigilance state, such as hand on the steering wheel, foot on the pedal and when driving in complex traffic conditions. A vigilance task was designed to simulate driver mental load in real-car driving in this study. This type of vigilance task has been confirmed to be suitable in simulating driving mental load (Noguchi et al., 1999). Figure 1 shows the protocol of the vigilance task. Three numbers changed randomly every second on the screen. The subjects need to immediately step on the brake pedal using right foot (i.e., within 1 s) when the three numbers are different odd numbers. The click time was recorded in real-time and the reaction time (RT) and accuracy were calculated. If the accuracy was below 80%, which indicates the participant did not maintain a high attention level, thus, this data was precluded. The task was performed in a silent room with a steering wheel, a brake pedal, and a computer screen, thereby simulating a car-driving environment. The participants were asked to put their hands on the steering wheel and maintain high attention.

Before the vigilance task, the subjects were required to keep the resting state for 10 min. Resting state is defined as a state when a subject is not performing an explicit task (Biswal, 2012). It is a natural condition in which there is no overt perceptual input or behavioral output. During resting state, the participants were instructed to sit comfortably in an adjustable chair, keep their eyes closed and stepped on the brake pedal. The subjects first assumed a sitting position to rest for 10 min. Thereafter, a 20-min task was performed immediately. The task was divided into two sessions for next analysis: the first 10 min (Task t1) and the second 10 min (Task t2). The NIRS measurement was performed during the whole experiment.

### NIRS Measurement

**Figure 2:**
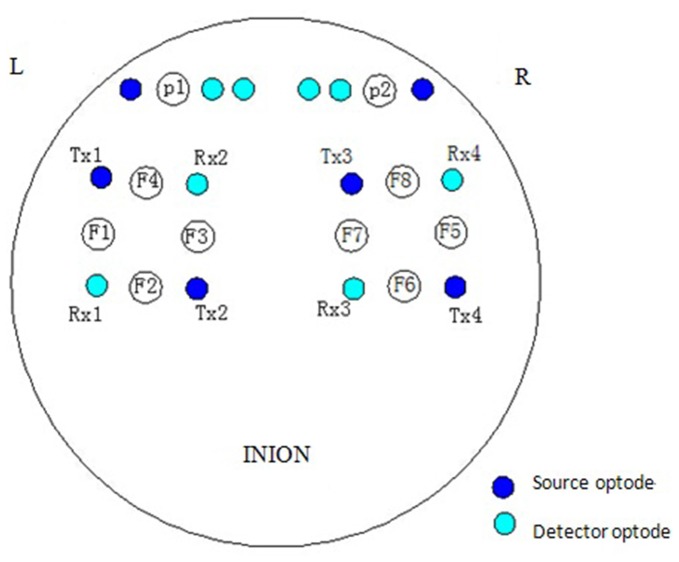
**Locations of source optode (blue dots) and detector optode (green dots).** A total of 10 channels were formed in the prefrontal cortex (PFC) and sensorimotor areas based on the international 10/10 system.

**Figure 3:**
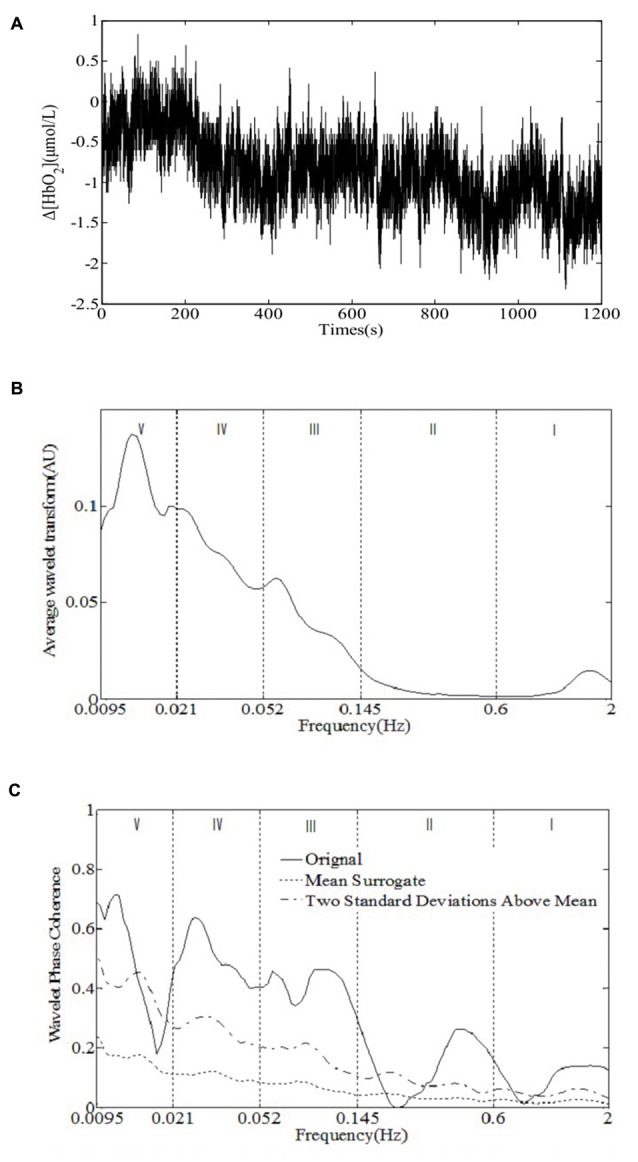
**An example of the raw time series Delta [HbO_2_] signals obtained from (A) the near-infrared spectroscopy (NIRS) measurements, (B) time-averaged wavelet transform amplitude, and (C) wavelet phase coherence (WPCO) of the two Delta [HbO_2_] signals.** The dashed and chain lines show the mean and two standard deviations (SD) above the mean, respectively, that is calculated from 100 surrogate signals obtained from the amplitude-adjusted Fourier transform (AAFT). The vertical lines indicate the outer limits of the frequency intervals: 0.6–2 Hz (I), 0.145–0.6 Hz (II), 0.052–0.145 Hz (III), 0.021–0.052 Hz (IV), and 0.0095–0.021 Hz (V).

The NIRS measurements were performed using a multi-channel tissue oxygenation monitor (TH200; developed by Tsinghua University, China) and eight-channel OXYMON MK III (Artinis Medical Systems B.V., Netherlands). The placement of the optodes has been described in our previous study (Tan et al., 2015). The sampling rate was set to 10 Hz. A sensor bandage was used to secure the sensor by wrapping it around the forehead to avoid any admission of background light. All the optodes of OXYMON MK III were inserted into a template holder. The template, which includes the optodes on it, was positioned over the sensorimotor cortical areas based on the international 10/10 system (Oostenveld and Praamstra, 2001). Figure 2 shows the location of the source optode (blue dots) and detector optode (green dots) in the PFC and sensorimotor areas. The Delta [HbO_2_] signals were monitored for 30 min in the frontal lobe and sensorimotor cortical areas. Figure 3A shows an example of raw time series Delta [HbO_2_] signals obtained from the NIRS measurements.

### Data Pre-Processing

First, the moving standard deviation (SD) method was used to remove the abrupt spikes in the raw Delta [HbO_2_] signals caused by movements and background light (Scholkmann et al., 2010). Second, a six-order Butterworth band-pass filter (0.0095–2 Hz) was used to remove the low variations (i.e., below 0.0095 Hz) and long-term baseline shifts. Third, the wavelet transform was calculated in the frequency range of 0.0095–2 Hz. Slow variations below 0.0095 Hz and uncorrelated components above 2 Hz were removed.

### Wavelet Transform

The wavelet transform is a method that provides appropriate time and frequency resolutions using adjustable filter band lengths. This method also provides a complex transformation of time series signals from the time to time–frequency domains to detect the linear combinations of the characteristic frequencies. The wavelet transform has been used in different types of physiological signals, such as skin blood flow oscillations and tissue oxyhemoglobin signals, among others (Stefanovska et al., 1999; Tan et al., 2015). A mother wavelet was used to detect the frequency content in a certain time interval. The time series was convolved with a family of generally non-orthogonal basis functions generated from the mother wavelet (Stefanovska et al., 1999; Bernjak et al., 2012). The resolutions of time (Δt) and frequency (Δω) were connected by Δ*t*Δω ≥ c, which was attained only for a Gaussian function (Kaiser, 2010). The Morlet wavelet is a Gaussian function that is modulated with a sine wave with basic frequency *ω_0_*. The Morlet wavelet is used because it provides good localization of events in both time and frequency domains (Bernjak et al., 2012). The current study can detect the frequency content of the signal by selecting *ω*_0_ = 2π to isolate the time content. Moreover, the frequency resolution is well enough in the case of *ω*_0_ = 2π (Stefanovska et al., 1999).

The cerebral NIRS signals, i.e., [HbO_2_], [dHb] and [tHb], recorded on a human head are considered to be composed of neurovascular coupling and systemic activity components (Scholkmann et al., 2014). The neurovascular coupling components include evoked neurovascular coupling by a stimulus/task, or non-evoked (i.e., spontaneous) neurovascular coupling (Scholkmann et al., 2014). As the entire cerebrovasculature is extensively innervated by adrenergic and cholinergic fibers of diverse extrinsic and intrinsic origins (Willie et al., 2014), a number of different, and possibly overlapping, physiological mechanisms such as the sympathetic nervous system, endothelial derived nitric oxide, and vascular myogenic responses could play some part in neurovascular coupling (Hamner et al., 2010). Five characteristic frequencies of skin blood flow signals corresponding to specific origins have been identified and confirmed in the human cutaneous circulation using wavelet analysis (Stefanovska et al., 1999; Shiogai et al., 2010). Similarly, the oscillations in cerebral NIRS signals with five characteristic frequencies have also been demonstrated in our previous studies (Li et al., 2010; Tan et al., 2015, 2016), which may reflect the neurovascular coupling and systemic regulation activities. Figure 3B shows an example of time-averaged wavelet transform amplitude of Delta [HbO_2_] signals obtained from the NIRS measurements. The periodic oscillations of Δ[HbO_2_] signals in five frequency intervals were identified: 0.6–2 Hz (I), 0.145–0.6 Hz (II), 0.052–0.145 Hz (III), 0.021–0.052 Hz (IV), and 0.0095–0.021 Hz (V). The frequency intervals were determined by the minima at each side of the peak.

### Wavelet Phase Coherence

WPCO identities possible relationships by calculating instantaneous phase difference of two signals. WPCO has a value between 0 and 1, which shows the tendency of phase difference between two signals at a certain frequency to remain constant (where the value would be close to 1) or unrelated (close to 0 because of the continuous changes of phase difference; Bernjak et al., 2012). Several physiological signals are characterized by high phase correlations and autocorrelation processes; thus, the surrogate method was adopted to avoid the influence of autocorrelation and to define the significance of phase correlation relationships (Sheppard et al., 2012). Significant coherence was tested by the evaluation of the coherence of two oscillatory time series, which may have variable amplitude and frequency. The test was performed by generating amplitude-adjusted Fourier transform (AAFT) surrogate signals via shuffling of the phases of the original time series to create a new time series with the same means, variances, and autocorrelation functions, which has the same power spectra as the original sequences. A temporally constant interference possibly exists between the signals regardless of their spectral similarities or differences when the coherence value is equal to the SDs above the mean surrogate coherence (Bernjak et al., 2012). It is considered statistically significant if an averaged WPCO value is above the mean surrogate with two SDs (Figure 3C).

The significant WPCO values were calculated as follows: Firstly, 100 AAFT surrogate signals (Bernjak et al., 2012; Tan et al., 2016) were generated to calculate the surrogate WPCO value. Secondly, the WPCO values and their surrogate signals were averaged across the subjects. Thirdly, the WPCO values were averaged across the interval. The average was performed with trapezoidal integral in each frequency intervals and divided it by the length of the frequency band. The presence of a significant WPCO value between two channels was considered to imply connectivity and marked with a blue line.

FC is usually characterized by a temporal correlation between two raw time series with low frequency (<0.1 Hz; Sasai et al., 2011), separable from respiratory (0.1–0.5 Hz) and cardiovascular (0.6–1.2 Hz) signal frequencies (Cordes et al., 2001). The systemic signals including respiratory and cardiovascular are commonly considered as global interference (Zhang et al., 2007). Therefore, the significant WPCO in intervals I and II formed the global connectivity (GC) maps and in intervals III to V formed the FC maps. The GC reflects the synchronization of cardiac and respiratory activities in the different cerebral areas, thereby demonstrating its difference from FC (Tan et al., 2015).

### Methodological Considerations

The NIR light must first pass through the superficial tissue layers (i.e., scalp and skull) before reaching the cortex. Therefore, these superficial layers may degrade the signal-to-noise ratio and nonspecific hemodynamic variations, thereby possibly contaminating the measured signal. The present study uses one light source and two detectors placed at 30 mm and 40 mm from the source to separate extracerebral (i.e., scalp and skull) and brain hemodynamics signals. Thus, the differences in the optical density (OD), as detected by the two detectors, were mainly attributed to the tissue (cortex) absorption.

Moreover, the null hypothesis that the coherence value is due to a chance relationship preserved over a limited number of correlated measurements must be considered when using the coherence to detect a causal relationship between signals (Sheppard et al., 2012). Low-frequency components are represented by fewer periods than high-frequency components in finite-length signals. Thus, limited variation in the phase difference occurs at low frequencies and results in artificially increased phase coherence.

### Data Analysis

The Δ[HbO_2_] signals obtained from four channels in the left sensorimotor areas were averaged as one single channel, which is the same as in the right sensorimotor areas. Thus, the four channels, which include two channels in the PFC, resulted in six channel pairs. The WPCO of each channel pair were calculated. The data of RT was recorded according to the difference between the time when the numbers appear and the time when the participants brake the pedal.

### Statistical Analysis

This study adopted the Kolmogorov–Smirnov test and Levene test to ensure that the values fulfilled the assumption required by the ANOVA analysis. *P* values for differences of RT between two period of the task (task t1 and task t2) were calculated using *t*-test for means and SDs. The Wilcoxon rank sum test was used to determine the significance level of the five frequency intervals of WPCO at the group level. One-way repeated ANOVA with interval as an independent variable was used to assess the main differences of WPCO between the resting and task condition. *Post hoc* analyses were performed using Bonferroni correction for multiple comparisons. A value of *p* < 0.05 was considered statistically significant.

## Results

**Figure 4:**
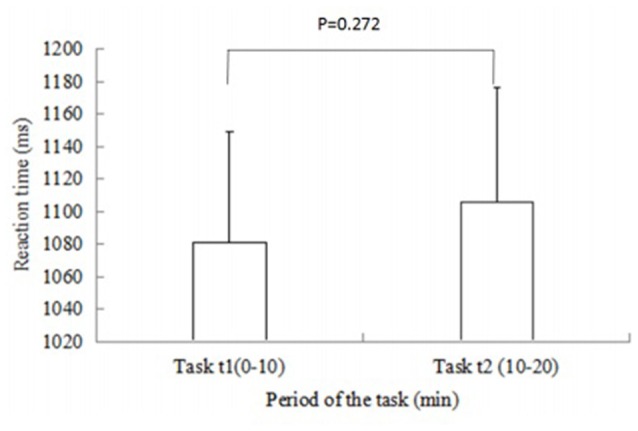
**Changes in mean reaction time (RT) in Task t1 and Task t2.** Vertical bars represent the SD. *P* values for differences are calculated using *t*-test for means and SDs. The RT shows an increase in Task t2 compared with that in Task t1. However, no significant difference was found between the two time periods.

Figure 4 shows the changes of mean RT during the task periods. *P* values for differences were calculated using *t*-test for means and SDs. The RT shows an increase in Task t2 compared with that in Task t1. However, this difference was not statistically significant between the two time periods (*p* = 0.272).

**Figure 5:**
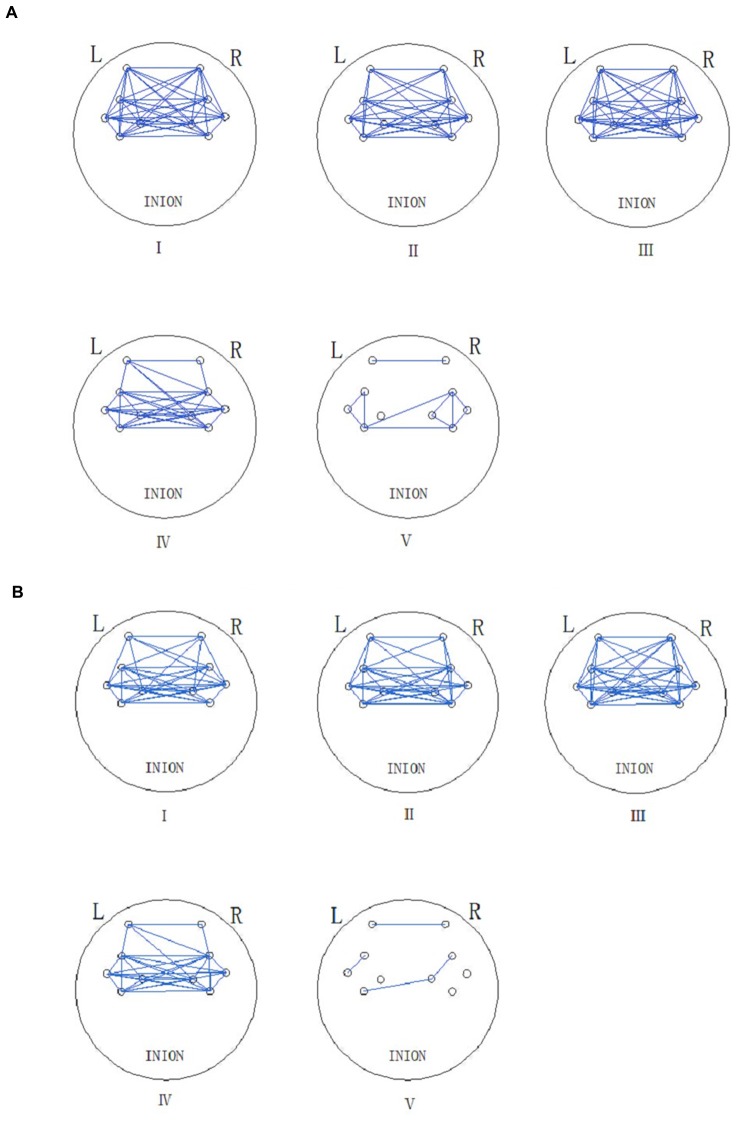
**Functional connectivity (FC; frequency intervals III–V) and global connectivity (GC; frequency intervals I and II) maps are revealed by the significant WPCO in the rest state (A) and task state (Task t2) (B).** The presence of a significant WPCO value between two channels was considered to imply connectivity and marked with a blue line. The five frequency intervals are 0.6–2 Hz (I), 0.145–0.6 Hz (II), 0.052–0.145 Hz (III), 0.021–0.052 Hz (IV), and 0.0095–0.021 Hz (V).

Figure 5 shows the connectivity maps revealed by WPCO in the five frequency intervals in the resting and Task (t2). The blue lines in the map indicate significant WPCO values. The connectivity maps showed compact connectivity (the ratio of the channels with significant WPCO values to total channels is higher than 50%) in intervals from I to IV and sparse connectivity in interval V among different brain regions. However, the map in interval I showed reduced connectivity between the prefrontal lobe and sensorimotor areas in Task t2, in which the GC decreased by 50% compared to resting state.

**Figure 6:**
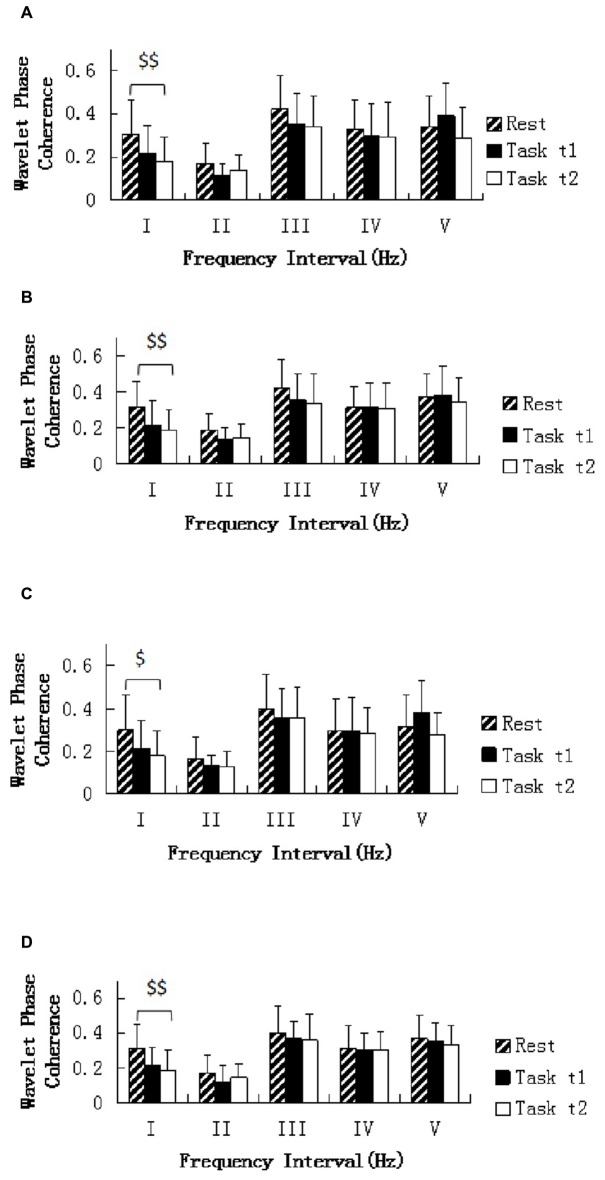
**Comparison of the WPCO value in the five frequency intervals between the left prefrontal regions and left sensorimotor area (A), between the left prefrontal regions and right sensorimotor area (B), between the right prefrontal regions and left sensorimotor area (C), and between the right prefrontal regions and right sensorimotor area (D).** Task t1 and Task t2 refer to the task at the first and second task sessions, respectively. Significant differences are marked with ^$^(*p* < 0.05) or ^$$^(*p* < 0.01) between the rest and task periods (i.e., Task t1 and Task t2, respectively). The five frequency intervals are 0.6–2 Hz (I), 0.145–0.6 Hz (II), 0.052–0.145 Hz (III), 0.021–0.052 Hz (IV), and 0.0095–0.021 Hz (V).

One-way repeated ANOVA revealed that the WPCO was significantly lower in Task t2 than at rest between left prefrontal and left sensorimotor cortex areas (*F* = 8.108, *p* = 0.007; Figure 6A), left prefrontal and right sensorimotor cortex areas (*F* = 10.705, *p* = 0.002; Figure 6B), right prefrontal and left sensorimotor cortex areas (*F* = 7.258, *p* = 0.01; Figure 6C), right prefrontal and right sensorimotor cortex areas (*F* = 9.044, *p* = 0.005; Figure 6D).

## Discussion

The current study measures the phase coherence between the Delta [HbO_2_] signals from different cortical areas during a sustained attention task using WPCO method. RT is characterized as a method to define the level of attention (Derosière et al., 2014). The RT results demonstrated a decreased attention level in Task t2 compared with that in Task t1.

The cerebral NIRS signals recorded on a human head are considered to be composed of neurovascular coupling and systemic activity components (Scholkmann et al., 2014). The systemic signals including respiratory and cardiovascular are commonly considered as global interference (Zhang et al., 2007). Cerebral response and systemic signals observed in functional near-infrared spectroscopy (fNIRS) and fMRI in the range of low frequency range between 0.1 Hz and 0.01 Hz are the basis of FC mapping (Sasai et al., 2011; Kirilina et al., 2012). Wu et al. (2008) demonstrated that the correlations among cortical networks are concentrated within ultra-low frequencies (0.01–0.06 Hz), while connections within limbic networks distribute over a wider frequency range (0.01–0.14 Hz). In the present study, the significant WPCO in intervals III–V (0.0095–0.145 Hz), which might reflect the neurovascular coupling activity, was defined as FC maps while significant WPCO in intervals I and II (0.145–2 Hz), which reflects the respiration and cardiac activities (systemic or global activities), defined as GC.

The wavelet-based coherence method decomposes time-series signals from the time domain into time–frequency domain. Several characteristic frequency intervals have been identified using wavelet analysis, which indicates the possible regulatory mechanisms of the cerebral tissue Delta [HbO_2_] signals (Li et al., 2010, 2013). The WPCO indicates the phase synchronization even at low common power (Grinsted et al., 2004; Bernjak et al., 2012; Han et al., 2014a; Tan et al., 2015, 2016). The high value of WPCO indicates a synchronization of Delta [HbO_2_] signals of different cortical areas.

This study showed a significant lower GC level in interval I in Task t2 than that at rest in the four channels pairs. The cardiac activity serves as a pump that drives blood through the vessels and the oscillation in interval I reflects this effect (Li et al., 2010; Shiogai et al., 2010). The level of WPCO in interval I reflects the synchronous contribution of cardiac activity to the Δ[HbO_2_] oscillations in the cerebral regions. As we know, the regulation of cerebral blood flow (CBF) is an integrative process that involves the marked influence of cardiovascular function (Willie et al., 2014). The brain controls the distribution of blood flow and redirects flow from other circulatory districts to the cerebral circulation through the humoral and neural influence over the cardiovascular system. When the activity of a brain region increases, CBF to that region also increases (Iadecola, 2004). This mechanism, termed functional hyperemia, controls substrate delivery and the removal of by-products of metabolism (Iadecola, 2004). In this study, a low WPCO value in interval I indicates a reduced phase synchronization of cardiac activity in the PFC and sensorimotor areas. The lower phase synchronization suggests a reduced coordinated regulation of cardiac activity to cerebral circulation between PFC and sensorimotor areas and this might affect substrate delivery and the removal of by-products of metabolism.

It has been demonstrated that strong FC existed among spontaneous fluctuations of the different brain regions in the low frequency during the resting state (Biswal et al., 1995; Zhang et al., 2010). FC between homologous regions in the contralateral hemisphere shows high coherence over a wide frequency interval (0.009–0.1 Hz; Sasai et al., 2011). The FC maps in the present study show a reduced FC in interval III in task state, particularly the connectivity between the left PFC and bilateral sensorimotor regions. The cerebral oscillation in interval III originated locally from the intrinsic myogenic activity of smooth muscle cells in the resistance vessels, which is under the autonomic control and associated with changes in the peripheral sympathetic nerve activity (Rowley et al., 2007; Shiogai et al., 2010). The present results indicate that the vigilance task leads to a decreased synchronization between the PFC and bilateral sensorimotor regions when subjects transitioned from rest to the task. Hermundstad et al. (2013) reported that the FC decreased during attention task but increased during memory task compared with that at rest. Fishburn et al. (2014) found increased FC with working memory load (n-back task) between frontal and parietal regions, between hemispheres for homologous frontal and parietal regions. This study confirmed that the FC would decrease in the attention state, and the significant decrease was only found in the latter half of the task (Task t2). This suggests that the change in GC at the range of 0.6–2 Hz was not attributed to the vigilance task *per se*, but the interaction effect of vigilance task and time factors. As indicated by the increased RT, the subject exhibited a low processing efficiency in Task t2. This may in turn contribute to the reduced GC.

A task-related decrease in synchronization between prefrontal and bilateral sensorimotor regions may be the result of a task-related increase of functional lateralization. An unconstrained resting state may have greater inter-hemispheric connectivity than a task that places demands on functions that are strongly lateralized. The lower phase synchronization suggests a reduced coordination between PFC and sensorimotor areas and this might impact task performance negatively.

Studies suggested that the cerebral oscillation in frequency IV might be regulated by neurovascular coupling and neurogenic activity on the vessel wall (Zhang et al., 2002). The neurogenic regulation allows the human body to adapt to a new environment based on internal or external factors. No significant changes of WPCO in interval IV were shown in this study. However, significant WPCO was determined more in interval IV between the bilateral sensorimotor areas than between the PFC and sensorimotor areas.

The oscillations in frequency interval V correspond to NO-related metabolic activity (Stefanovska et al., 1999) and this metabolic regulation facilitates blood flow to satisfy the need for cell oxygen (Humeau et al., 2004). Interestingly, the WPCO in interval V (0.0095–0.021 Hz) between the left PFC and left sensorimotor areas, as well as the right PFC and left sensorimotor areas, exhibited a higher level in Task t1 than in the resting state. The higher WPCO between the PFC and left sensorimotor areas suggest an enhanced synchronization of metabolic activity in response to the vigilance task.

## Conclusion

This study demonstrated that the GC levels in interval I was significantly lower in the task state than in the resting state. Significant difference was found only between resting state and later half of the task. The results showed that the change in FC at the range of 0.6–2 Hz was not attributed to the vigilance task *per se*, but the interaction effect of vigilance task and time factors. The findings suggest that the decreased attention level might be partly attributed to the reduced GC levels between the left prefrontal region and sensorimotor area. The present results provide a new insight into the vigilance task-related brain activity.

## Author Contributions

ZL designed the study and edited the manuscript. WW did the experiment, analyzed the data and drafted the manuscript. BW designed the vigilance task. LB did the experiment and analyzed the data. LX performed the statistical analysis. YF contributed to the physiological interpretation of the results and edited the manuscript.

## Conflict of Interest Statement

The authors declare that the research was conducted in the absence of any commercial or financial relationships that could be construed as a potential conflict of interest.
